# High-speed classification of coherent X-ray diffraction patterns on the K computer for high-resolution single biomolecule imaging

**DOI:** 10.1107/S0909049513022152

**Published:** 2013-10-03

**Authors:** Atsushi Tokuhisa, Junya Arai, Yasumasa Joti, Yoshiyuki Ohno, Toyohisa Kameyama, Keiji Yamamoto, Masayuki Hatanaka, Balazs Gerofi, Akio Shimada, Motoyoshi Kurokawa, Fumiyoshi Shoji, Kensuke Okada, Takashi Sugimoto, Mitsuhiro Yamaga, Ryotaro Tanaka, Mitsuo Yokokawa, Atsushi Hori, Yutaka Ishikawa, Takaki Hatsui, Nobuhiro Go

**Affiliations:** a RIKEN SPring-8 Center, 1-1-1 Kouto, Sayo-cho, Sayo-gun, Hyogo 679-5148, Japan; b Department of Computer Science, Graduate School of Information Science and Technology, The University of Tokyo, 7-3-1 Hongo, Bunkyo-ku, Tokyo 113-0033, Japan; c JASRI, 1-1-1 Kouto, Sayo-cho, Sayo-gun, Hyogo 679-5198, Japan; d System Software Research Team, Research Division, RIKEN Advanced Institute for Computational Science, 7-1-26 Minatojima-minami-machi, Chuo-ku, Kobe, Hyogo 650-0047, Japan; e Operations and Computer Technologies Division, RIKEN Advanced Institute for Computational Science, 7-1-26 Minatojima-minami-machi, Chuo-ku, Kobe, Hyogo 650-0047, Japan; f Molecular Modeling and Simulation Group, Japan Atomic Energy Agency, 8-1-7 Umemidai, Kizugawa, Kyoto 619-0215, Japan

**Keywords:** X-ray free-electron laser, K computer, single-particle coherent diffraction imaging, classification of diffraction patterns, big-data analysis

## Abstract

Single-particle coherent X-ray diffraction imaging using an X-ray free-electron laser has the potential to reveal the three-dimensional structure of a biological supra-molecule at sub-nanometer resolution. In order to realise this method, it is necessary to analyze as many as 1 × 10^6^ noisy X-ray diffraction patterns, each for an unknown random target orientation. To cope with the severe quantum noise, patterns need to be classified according to their similarities and average similar patterns to improve the signal-to-noise ratio. A high-speed scalable scheme has been developed to carry out classification on the K computer, a 10PFLOPS supercomputer at RIKEN Advanced Institute for Computational Science. It is designed to work on the real-time basis with the experimental diffraction pattern collection at the X-ray free-electron laser facility SACLA so that the result of classification can be feedback for optimizing experimental parameters during the experiment. The present status of our effort developing the system and also a result of application to a set of simulated diffraction patterns is reported. About 1 × 10^6^ diffraction patterns were successfully classificatied by running 255 separate 1 h jobs in 385-node mode.

## Abstract 2

A code with an algorithm for high-speed classification of X-ray diffraction patterns has been developed. Results obtained for a set of 1 × 10^6^ simulated diffraction patterns are also reported.

## 1. Introduction

The X-ray free-electron laser (XFEL) generates an intense X-ray laser pulse as short as a few femtoseconds. This type of light source is anticipated to offer a new possibility of single-particle coherent X-ray diffraction imaging (CXDI) for non-crystalline biomolecular samples (Neutze *et al.*, 2000 ▶; Schlichting & Miao, 2012 ▶). The intense X-ray laser pulse is irradiated onto a single biomolecular target, and two-dimensional coherent diffraction patterns are recorded repeatedly, each for a random unknown orientation. Even with the use of an intense XFEL, the diffraction intensity arising from a single particle is weak, causing diffraction patterns deeply immersed in quantum noise.

A decade ago, a basic scheme of data analysis for three-dimensional structure determination was suggested (Huldt *et al.*, 2003 ▶). This scheme consists of three steps. At first, the diffraction patterns are classified according to similarity and averaged within each similarity group in order to improve the signal-to-noise (S/N) ratio. Then, a three-dimensional diffraction intensity function is constructed by aligning signal-enhanced two-dimensional diffraction patterns in reciprocal space. Finally, the phase is retrieved by applying the over-sampling method (Sayre, 1952 ▶; Gerchberg & Saxton, 1972 ▶; Fienup, 1982 ▶).

Generally, along the suggested line, we reported a detailed algorithm for classifying and assembling two-dimensional noisy diffraction patterns to construct a three-dimensional diffraction intensity function (Tokuhisa *et al.*, 2012 ▶). The algorithm enables signals immersed in the quantum noise to be extracted, which is indispensable in constructing a near-atomic-resolution three-dimensional structure. We have reported that the algorithm can classify the diffraction data with statistics as low as 0.1 photons pixel^−1^. To classify diffraction patterns according to the similarity, a correlation pattern is calculated for each pair of diffraction patterns. In order to construct a structure with sub-nanometer resolution for the case of 70S ribosome, it is necessary to analyze about 1 × 10^6^ diffraction patterns.

All-pair calculation for this number of patterns is of high computational cost. In this paper we report a *representative-all pair scheme* in order to reduce the cost significantly. In this scheme, correlation patterns are calculated between one from two-dimensional diffraction patterns representing each similarity group and the other from a set of whole observed two-dimensional diffraction patterns. The number of correlation patterns to be calculated is about 13 billion for the above example. Even with this representative-all pair scheme, the calculations take about 100 days in the case of a 10TFLOPS computer.

For a system of data analysis to be practically useful, it is necessary to process the calculation concurrent to the data collection in order to diagnose the data quality during the experiments. The calculation results can then be used to optimize the experimental parameters (Tokuhisa, 2013 ▶). To achieve these goals we have implemented a code of high-speed classification on the K computer, a 10PFLOPS supercomputer at RIKEN Advanced Institute for Computational Science (AICS; http://www.aics.riken.jp/en/). We report the present status of our developments on (i) the non-visual automatic similarity detection algorithm, (ii) the representative-all pair classification scheme, (iii) program parallelization, and (iv) an efficient diffraction data flow between the XFEL facility, SACLA (Ishikawa *et al.*, 2012 ▶), and the K computer. Computation results obtained using a set of 1 × 10^6^ simulated diffraction patterns are also reported.

## 2. A high-speed classification system

### 2.1. Automatic similarity detection algorithm

In our method of detecting similarity between a pair of two-dimensional diffraction patterns *i* and *j*, we calculate a correlation pattern *c*_*ij*_(ξ,α) as a function of two variables ξ and α and defined as follows (Tokuhisa *et al.*, 2012 ▶),
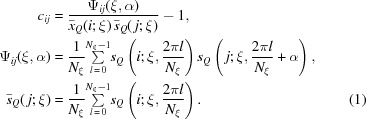
Here ξ is the angle of diffraction, which is expressed as 2θ in the usual literature, α is the angle of rotation of the detector plane around the incident beam axis, *N*_ξ_ is the number of Shannon pixels on a circle with a fixed value of ξ, *s*_*Q*_ is the photon number to be observed by a detector Shannon pixel with solid angle ω, and 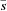 is its mean over pixels on the above circle. The quantum-mechanically expected mean *s*(**k**) of *s*_*Q*_ is given by
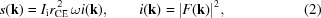
where *I*_i_ is the incident X-ray intensity, *r*_CE_ is the classical electron radius, *F*(**k**) is the structure factor, **k** is the momentum transfer and *i*(**k**) is the diffraction intensity density. The magnitude of momentum transfer is given as
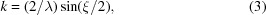
where λ is the wavelength of the incident X-ray.

**Figure 1:**
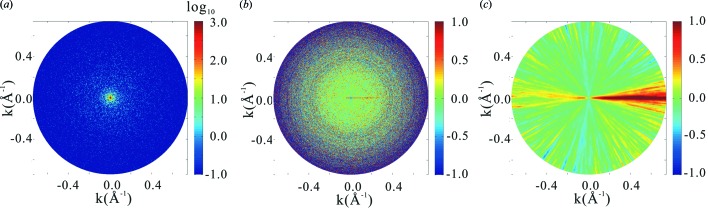
(*a*) Simulated diffraction pattern *s*_*Q*_ with quantum noise for the 70S ribosome by assuming the incident X-ray intensity to be *I*_i_ = 2.55 × 10^20^ photons pulse^−1^ mm^−2^. (*b*) Correlation pattern *c*_*ij*_ for a pair of between diffraction patterns *i* and *j*. (*c*) Integrated correlation pattern *I*_c_.

Simulated examples of *s*_*Q*_ and *c*_*ij*_ are shown in Fig. 1 ▶. Reflecting the fact that the target is a single particle, the experimentally observed diffraction pattern *s*_*Q*_(ξ,α) is immersed deeply in the quantum noise especially in the higher-angle range. This noisy nature of *s*_*Q*_ is inherited in the noisiness of *c*_*ij*_. When a pair of *s*_*Q*_s for *i* and *j* are similar, a high correlation line appears in *c*_*ij*_. The correlation line becomes invisible against the noisy background at a high *k* region, *k* > *k*_N_, where *k*_N_ (subscript standing for ‘noise’) is the value of *k* at which the standard deviation of the background noise becomes as high as 0.6 ≃ exp(−1/2) (Tokuhisa *et al.*, 2012 ▶).

Detection of similarity between a pair of *s*_*Q*_s is thus translated to detection of a high correlation line in *c*_*ij*_. To do this job at high speed, we developed an algorithm of non-visual automatic similarity detection. The basic idea of the algorithm is to use the following integral value of *c*_*ij*_ so that the quantum noise is averaged out within a single figure and the positive definite signals are enhanced by integration,
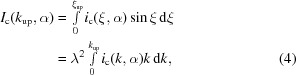


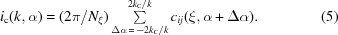
Here, *k*_C_ is the correlation length of the intensity data which is approximately given by *k*_C_ = 1/*L* with *L* being the length of a sample molecule. In our method, the upper bound of the integration *k*_up_ is chosen in the range *k*_up_ ≤ *k*_*N*_ where 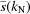 = 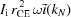 assumes the value of about 0.1. An example of this integrated correlation pattern, *I*_c_, is also shown in Fig. 1 ▶. If a significant maximum of *I*_c_(*k*_up_,α) is detected at α = 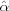, it gives the direction of the high correlation line. We then identify the value of *k*_up_ for which *I*_c_(*k*_up_,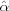) assumes the peak value within the range *k*_up_ ≤ *k*_N_, write such a value as 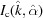, and will refer to it as the peak value of the integrated correlation. This value is used to judge the similarity between the pair of *s*_*Q*_s for *i* and *j*. A higher value of 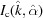 means a higher similarity. Use of *I*_c_ contributed to improve the sensitivity of the method significantly.

In our method, *s*_*Q*_s are classified according to similarity and averaged within each similarity group. The similarity is judged by 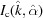. If we employ a higher threshold value of 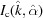 for a pair of *s*_*Q*_s to be classified into one group, the number of similarity groups will become larger. But, at the expense of a large number of groups, we can attain higher structural resolution of the final result. We can control the obtainable structural resolution by the threshold value of 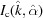.

### 2.2. Representative-all pair classification scheme

To avoid the necessity of carrying out *c*_*ij*_ calculations for all pairs of 1 × 10^6^ *s*_*Q*_s, we adopt the *representative-all pair scheme* mentioned in the *Introduction* [Sec sec1]. This scheme contains two tasks: (i) selection of representative *s*_*Q*_s and (ii) implementation of similarity detection for pairs, each pair consisting of one from a set of the representative *s*_*Q*_s and the other from the set of whole *s*_*Q*_s. Even though it is conceivable to design a scheme to solve task (i) while processing task (ii), we nonetheless developed a simple scheme in which the two tasks are carried out in two separate steps in sequence. This simple scheme has the merit of being quick and flexible. Because of its quickness, this scheme is suited for the real time application of the analysis system.

In step 1, task (i) is carried out as follows. We start from defining a targeted structural resolution. In the simulation calculation for 70S ribosome, a particle with length *L* of about 270 Å, we set the targeted resolution *r* to be about 5 Å. This value is translated to an allowed solid angle ω_G_ = 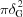 of a circular disc on the Ewald sphere for each similarity group, where δ_G_ = *r*/*L* (Tokuhisa *et al.*, 2012 ▶) turns out to be about 1°. In order to select a good set of representative *s*_*Q*_s, the respective beam directions of the associated patterns should not be close. Here we select a set of representative *s*_*Q*_s that satisfies all the angles between the respective beam directions larger than δ_G_ on the Ewald sphere. We estimate the maximum number of points on the sphere satisfying this requirement by 4π/ω_G_, where 4π is the solid angle of the whole sphere. This number turns out to be about 13000. Thus the targeted resolution is translated to the number of representative *s*_*Q*_s. We then prepare a set of relatively small number of *s*_*Q*_s sampled from uniform random orientations for which all pair *c*_*ij*_ calculation is possible. In our simulation we prepared tentatively a set of about 1.5 times as many diffraction patterns as compared with the number of targeted representative *s*_*Q*_s. It should be noted that such a set of relatively small number of *s*_*Q*_s can be prepared at an early stage of an on-going experiment. For this small set we carried out all pair *c*_*ij*_ calculations to obtain 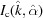. By referring to 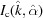 sorted in descending order, we identify pairs of *s*_*Q*_s in each of 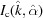, and erase one of the pair of *s*_*Q*_s in this order from the list of candidates of representatives until the remaining number of candidates becomes exactly the number of targeted groups. 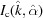, where this occurs, is recorded as the threshold peak value *I*_c,representative_ to be referred to in task (ii).

In step 2, task (ii) is carried out as follows. At first 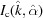 is calculated for all pairs, each pair consisting of one from the representative *s*_*Q*_s and the other from the whole set of about 1 × 10^6^ *s*_*Q*_s. This part of the calculation can be divided into independent separate jobs by dividing the large set of whole *s*_*Q*_s into subsets; or, even while the whole set is being generated during the experiment, calculation can be started for a part of the growing set. This flexible feature is a result of the two-step scheme we adopted.

As a result of step 2, about 80 *s*_*Q*_s on average are expected to be assigned to belong to each similarity group. This is the number needed to improve the S/N ratio so that mutual alignment of signal-enhanced *s*_*Q*_s in the reciprocal space can be performed.

After the correlation calculations, we proceed to identify pairs judged to be similar. This is done by comparing 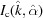 for each pair with a certain threshold value of *I*_c,group_. In this paper, values of *I*_c,group_ were chosen so that the average number of *s*_*Q*_s in the similarity groups is larger than 80 and *I*_c,group_ < *I*_c,representative_.

### 2.3. Assessment of the algorithm

The algorithm we propose in this paper carries out similarity detection among a large number of experimentally observed diffraction patterns. The algorithm also gives a relative rotation angle α_*ij*_ of the detector plane for each pair. A pair of *s*_*Q*_s for *i* and *j* is defined to be similar, when the angle β_*ij*_ between the respective beam directions is less than a certain cut-off value β_0_. We are applying the proposed algorithm for a set of *s*_*Q*_s. Because *s*_*Q*_s in this paper are the simulated diffraction patterns, the values of α_*ij*_ and β_*ij*_ are in fact known precisely beforehand. We can assess the quality of the proposed algorithm by comparing its result with precise values from the simulation.

The result of assessment is expressed in terms of two probabilities, *P*_right_, the probability that a result of classification is right, and, *P*_capture_, the probability that a right pair is captured, which are expressed, respectively, as follows, 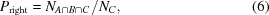


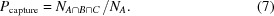
Here the quantities appearing on the right-hand-sides are defined as follows. A set of pairs whose simulation β_*ij*_ values are smaller than a certain value β_0_ is defined as *A* with its number of elements denoted as *N*_*A*_. Set *B* is defined as a set of pairs whose 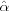 value is within such a narrow range around α_*ij*_ as 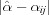 < 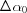, where 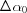 is a small value of angle taken to be 1° in this paper. A set of pairs that are judged by the algorithm to have high 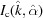 is defined as *C* with its number of elements denoted as *N*_*C*_. A set of pairs that are correctly captured and judged as a right pair is given by the product set 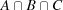 with its number of elements designated as 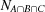.

### 2.4. Parallelization of the classification program

Basically, the correlation calculation must be applied to any possible combination of two *s*_*Q*_s. This procedure can be easily parallelized by decomposing the diffraction data set; however, a naïve implementation cannot avoid reading the same file multiple times and this file I/O can be a severe performance bottleneck.

The K computer consists of 82944 nodes. Since each node has 16 GB of memory, the total amount of memory of the K computer is approximately 1.3 PB. The total size of 1 × 10^6^ diffraction patterns is approximately 14 TB, much smaller than the whole memory size of the K computer. Thus, all diffraction data can be loaded into the memory of the K computer. The first prototype program was developed by using the MPI (Message Passing Interface) library. Each MPI process reads a dedicated file and then the read data is passed to the other nodes upon request. In this way, the file I/O bottleneck can successfully be avoided. Based on this prototype, a new program is under development to achieve better performance.

## 3. Result of application of developed classification scheme

<!?tpt=-4.5pt>In this section, we report the result of application of the developed scheme and algorithm for diffraction data simulated for 70S ribosome. The incident X-ray wavelength λ = 1 Å and intensity *I*_i_ = 2.55 × 10^20^ photons pulse^−1^ mm^−2^ are assumed in the simulation. This intensity can be realised when the XFEL beam emitted at SACLA is fully transported and focused down to 50 nm × 50 nm. Note that this focusing condition will make the hit rate of the XFEL pulse to the molecule lower and may require novel experimental methodology. For this molecule, we set the targeted resolution *r* to be 4.7 Å. This value corresponds exactly to δ_G_ being 1.0°. This resolution is translated to the number of similarity groups to be 13146 and to the necessary number of *s*_*Q*_s to be 1.05 million. In our treatment in this paper we do not explicitly pay attention to the centrosymmetric property of *i*(**k**). When we take this symmetry into account (which we should in real experiments), a single *s*_*Q*_ is to be subjected to the classification twice, the first time as the pattern itself and the second time as its centrosymmetric pattern. In this treatment, 1.05 million *s*_*Q*_s for classification can be prepared from the half number of *s*_*Q*_s (Tokuhisa *et al.*, 2012 ▶). In this paper we prepared 1.05 million *s*_*Q*_s by using equation (2) and the PDB coordinate of 70S ribosome, 1yl3 and 1yl4 (Jenner *et al.*, 2005 ▶). Each *s*_*Q*_ consists of photon-count data by pixels arranged in a two-dimensional square lattice. The photon-count data are given up to the diffraction angle corresponding to 0.74 Å^−1^, a value far enough to achieve 4.7 Å resolution. These *s*_*Q*_s on a square lattice were then converted into a form suitable for the *c*_*ij*_ computation, *i.e.* photon-count data by fictitious pixels on a circle with fixed value of ξ. In fact, in order to compute *c*_*ij*_ rapidly using a fast Fourier transform library, a Fourier transform of such data is prepared and stored in place of the original *s*_*Q*_. The size of the data for the whole 1.05 million *s*_*Q*_s is 14 TB.

In step 1 for selection of the representative *s*_*Q*_s, 13252 patterns (slightly different from the targeted number 13146 for a very technical reason) were selected from a set of exactly 20000 *s*_*Q*_s by the method described in §2.2. The obtained value of *I*_c,representative_ is 0.00111. The distance to the nearest representative is found distributed roughly between 0.4 and 2.2° with the average being 1.1°, which is very near to our target value of 1.0°. The result shows that our algorithm can detect the similarity between a pair of *s*_*Q*_s with an accuracy of about 1°. The calculation of this step was carried out in one job using the computational resource of 3.8 M nodes s.

The calculation of *I*_c_ for classification of 1.05 million diffraction patterns into 13252 groups was carried out by dividing the whole calculation into 255 independent 1 h jobs, each using 385 nodes, with the total computational resource used being 207 M nodes s. The total number of correlation calculations is thus 13.8 billion. The input data for each job is (i) the data set of all representative *s*_*Q*_s, common for all jobs, and (ii) a part of the data set from the whole *s*_*Q*_s allocated to the job. If we use all of the 82944 nodes of the K computer, the whole calculation can be finished in 71 min.

**Figure 2:**
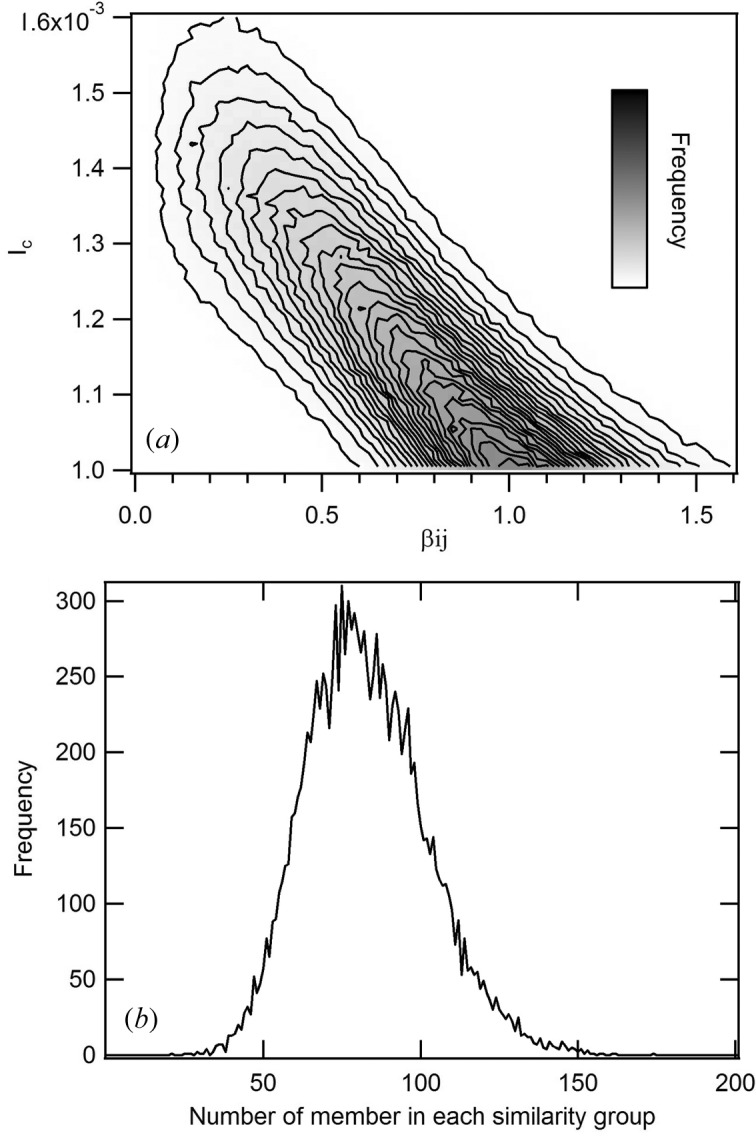
Result of classification of a set of about 1 × 10^6^ diffraction patterns for 70S ribosome obtained by simulation assuming the intensity of incident X-ray is *I*_i_ = 2.55 × 10^20^ photons pulse^−1^ mm^−2^. (*a*) Distribution of values of 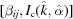, where pairs with 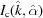 > *I*_c,group_ = 0.0010 are judged similar and β_*ij*_, the angle between each incident beam direction for a pair, is the value known from the simulations. (*b*) Distribution of the number of members in each similarity group.

After calculation of 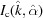 for all *representative-all* pairs, we proceed to judge whether or not each *s*_*Q*_ from the whole set belongs to the similarity group of each representative. This judgement is done by comparing 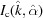 for each pair with a threshold value, *I*_c,group_, for the judgement. In this paper, *I*_c,group_ is set to be 0.0010 as described in §2.2, yielding the average number of *s*_*Q*_s in the similarity groups to be 82.8. Here we allowed one *s*_*Q*_ to belong to more than one group. In this treatment, a total of 1097672 pairs are assigned as similar (set *C*). For each pair thus judged to be similar, the exact value of similarity β_*ij*_ is in fact known from the record of simulation. The distribution of the similarity value β_*ij*_ *versus* *I*_c_ is shown in Fig. 2(*a*) ▶. Almost all β_*ij*_ are less than 2.0°, indicating that the attainable resolution of our analysis is better than 9.4 Å. This shows that our method can achieve sub-nanometer-resolution three-dimensional imaging of biomolecules with an XFEL. For higher-resolution imaging, we should solve a few problems. It is noted that about 28% of *s*_*Q*_s were found to be orphans, *i.e.* to belong to no similarity groups of the representatives. Upgrading of the method for the selection of the representatives should reduce the number of orphans. Our algorithm failed to identify 133387 pairs with β_*ij*_ < 1° as belonging to set *C*. Out of the pairs in set *C*, 713248 pairs are found to belong to set 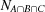. *P*_right_ and *P*_capture_ are found to be 0.65 and 0.68, respectively. Revision of the automatic similarity detection method, *e.g.* equation (4), should improve these values.

Fig. 2(*b*) ▶ shows the distribution of the number of *s*_*Q*_s classified in each similarity group. In cases where the classification calculation is carried out on a real-time basis, the diffraction pattern collection experiment should be carried out by monitoring such a graph as in Fig. 2(*b*) ▶ until the average becomes larger than 80.

## 4. An efficient data flow between SACLA and the K computer

**Figure 3:**
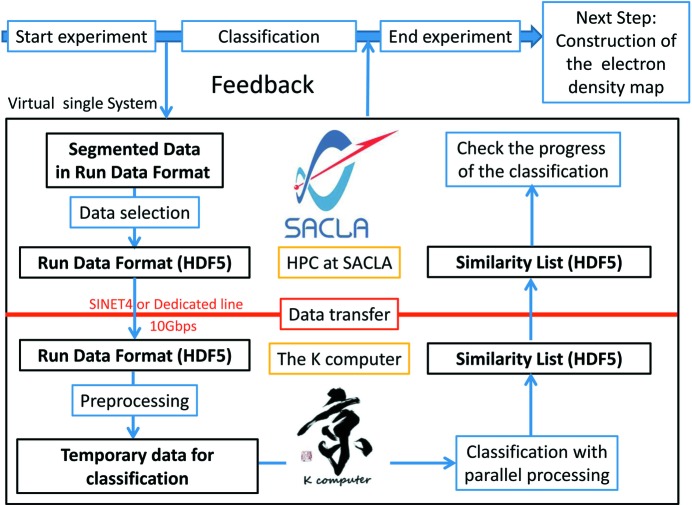
Schematic diagram showing an efficient data flow between the XFEL facility SACLA and the K computer.

The SACLA facility is located 60 km in a straight line from the K computer. Both facilities are connected *via* the Wide Area Network, SINET4 (http://www.sinet.ad.jp/index_en.html). The data transfer system is now under construction. In Fig. 3 ▶ we show the data flow diagram. First, the diffraction data are saved to a storage device in SACLA in a run data format. Next, *s*_*Q*_s not suitable for analysis are excluded by applying a filtering algorithm. Data sets of *s*_*Q*_s, each with a proper size, are then transferred from SACLA to the K computer in a certain interval by using SINET4, where 10 Gbps bandwidth is reserved from the SACLA facility to the edge node of SINET4. A dedicated network is also in the proposal phase to secure the on-line data-transmission bandwidth. In the K computer, each *s*_*Q*_ is then converted into a Fourier-transformed format suitable for subsequent calculation of *c*_*ij*_ before the two-step classification computation is executed. During the classification calculations, the temporal results can be monitored remotely from the SACLA beamline endstation so that data quality can be diagnosed by the experimentalists. The run data format and the similarity list are implemented on HDF5 (HDF group, http://www.hdfgroup.org/). The complete system of the above data flow will be operational in the near future.

## 5. Conclusion

We developed a code with a classification algorithm (Tokuhisa *et al.*, 2012 ▶) compatible with data as large as 1 × 10^6^ diffraction patterns. The code is designed so as to be able (i) to finish the whole classification calculation within about 1 h of computation by the K computer, and (ii) to conduct the classification concurrent to the experimental data collection. The benchmark with simulated data demonstrated the speed and flexibility that enables the target experimental scheme. It is shown that our method can achieve a sub-nanometer resolution imaging by the synergistic use of SACLA and the K computer.

We have found a rather large number (about 28%) of diffraction patterns (orphans) which were not classified into any similarity groups. An *ad hoc* improvement would be to select additional representatives from the orphans, and re-calculate grouping for those additional representatives. A more serious improvement would be to re-examine the estimation of the number of representative diffraction patterns by 4π/ω_G_ and the size of a relatively small set of diffraction patterns (currently 1.5 times the targeted number) from which the targeted number of representatives are selected. We expect that the above change would also contribute to improve the observed *P*_right_ and *P*_capture_. Revision of the automatic similarity detection method is also under consideration for the improvement of these quantities. Use of a high-performance I/O library will reduce the reading and the writing time of the data which occupy half of the execution time in this work.

Recently, illumination of multiple molecules to increase the scattering intensity has been proposed to overcome the low statistics of each diffraction pattern (Oroguchi & Nakasako, 2013 ▶). In this case, the analysis of the diffraction patterns becomes more complex, and makes the attribution of diffraction patterns to the structure of each molecule limited. On the other hand, single-particle coherent X-ray imaging, which has been discussed in this paper, has a clear physical relation between the diffraction pattern and the structure of each molecule. The latter has several technological issues to be overcome, such as a low hit rate of particles by the XFEL pulse. One of them is the diagnostics of the data quality. The present study shows that data diagnostics during the data acquisition can be executed by the dedicated code implemented on the state-of-art computation infrastructure.

## References

[bb1] Fienup, J. R. (1982). *Appl. Opt.* **21**, 2758. 10.1364/AO.21.002758 20396114

[bb2] Gerchberg, R. W. & Saxton, W. O. (1972). *Optik*, **35**, 237–246.

[bb3] Huldt, G., Szőke, A. & Hajdu, J. (2003). *J. Struct. Biol.* **144**, 219–227. 10.1016/j.jsb.2003.09.025 14643224

[bb4] Ishikawa, T. *et al.* (2012). *Nat. Photon.* **6**, 540–544.

[bb5] Jenner, L., Romby, P., Rees, B., Schulze-Briese, C., Springer, M., Ehresmann, C., Ehresmann, B., Moras, D., Yusupova, G. & Yusupov, M. (2005). *Science*, **308**, 120–123. 10.1126/science.1105639 15802605

[bb6] Neutze, R., Wouts, R., van der Spoel, D., Weckert, E. & Hajdu, J. (2000). *Nature (London)*, **406**, 752–757. 10.1038/35021099 10963603

[bb7] Oroguchi, T. & Nakasako, M. (2013). *Phys. Rev. E*, **87**, 022712. 10.1103/PhysRevE.87.022712 23496553

[bb8] Sayre, D. (1952). *Acta Cryst.* **5**, 843.

[bb9] Schlichting, I. & Miao, J. (2012). *Curr. Opin. Struct. Biol.* **22**, 613–626. 10.1016/j.sbi.2012.07.015 PMC3495068 22922042

[bb10] Tokuhisa, A. (2013). *Housyakou*, **26**, 26–37. (In Japanese.)

[bb11] Tokuhisa, A., Taka, J., Kono, H. & Go, N. (2012). *Acta Cryst.* A**68**, 366–381. 10.1107/S010876731200493X PMC3329770 22514069

